# Climate change and health: An assessment of state level adaptation plans

**DOI:** 10.1016/j.joclim.2025.100593

**Published:** 2025-10-22

**Authors:** Katharine Weber, Aparna Bole, John Balbus

**Affiliations:** Katharine Weber, Physician, Affiliate Faculty University of Colorado Aparna Bole, Adjunct Associate Professor, Pediatrics, Case Western Reserve University

**Keywords:** Climate, Health, States, Adaptation, Planning, Federal government

## Abstract

**Introduction:**

While health impacts of climate change are increasingly evident, adaptation planning for climate health impacts in the United States (US) has lagged. In the absence of a national climate and health adaptation plan, varied approaches have been taken by states to address health in their adaptation planning. The authors reviewed state adaptation plans developed since 2008 to assess how health adaptation strategies were included and to document identified adaptation gaps and needs.

**Methods:**

Plans were identified through Georgetown Climate Center’s State Adaptation Progress Tracker and a Google search. The authors developed a scoring rubric for consistency and evaluated plans based on such criteria as: Comprehensiveness, Inclusiveness, Monitoring and Evaluation. Adaptation priorities were noted and mapped to six categories.

**Results:**

19 plans met inclusion criteria. Most plans (14) identified exposure pathways impacting health. About half of plans identified vulnerable populations, but only three addressed the vulnerability of health systems. Most plans (13 of 19) did not mention a vulnerability assessment or cite a data source discussing vulnerability indicators. Only two plans had clear metrics for success. Only three mentioned an implementation timeline.

**Conclusion:**

This review highlights both positive aspects and gaps in state climate and health planning. Many state plans did discuss climate and health, exposure pathways, and vulnerable populations. States lack clear metrics for monitoring and evaluation or implementation. States may benefit from federal leadership through a national-level climate and health adaptation plan or the federal government’s development of planning guidance for states, localities, tribes and territories.

## 1 Introduction

It is clear that climate change is already affecting the health of people in the United States [1,2]. While adaptation strategies can reduce the health impacts of climate change, the development of climate change adaptation strategies in general, and comprehensive climate change and health adaptation strategies specifically, has been lagging in the United States [1,3]. At present, the United States, unlike several other nations, lacks a comprehensive National Adaptation Plan or analogue [[4], [5], [6]].

While the U.S. Centers for Disease Control and Prevention has supported state level climate adaptation activities, the focus has been on risk and vulnerability assessment and specific adaptation interventions, rather than comprehensive state level planning [7]. The US federal government does not provide a planning template, set of criteria or guidance for a comprehensive state climate adaptation plan for health or similar materials for localities, tribes or territories [8]. According to the 5th National Climate Assessment, between 2018 and 2022, 18 states had completed state-level adaptation plans and six had plans in process, though it was not specified whether health was included in the plans [3].

Because of the federal system of governance in the United States, states have considerable discretion in the governance of their healthcare and public health systems [9,10]. Broadly speaking, federal agency roles focus on financing and providing general guidance and at times establishing baseline standards for major health and public health programs, while states implement those programs, fund health care systems, and pass through some financial support to local agencies (e.g., county, city, town/village). Thus, to inform the national agenda for climate change adaptation for health, it is critical to identify gaps and priority needs at the state, local, tribal and territorial levels.

Accordingly, as a starting point, the authors conducted a scoping review of state adaptation plans for climate change and health, with the intent to systematically identify gaps and priority needs articulated by state-level agencies and stakeholders. Recognizing a high degree of variability in comprehensiveness and approaches taken among the plans, the authors developed a scoring rubric to assess these plans’ strengths and weaknesses. This paper describes the scoring rubric, provides the results of assessing each identified state climate adaptation plan for health against this rubric, and identifies themes in the priority needs and gaps mentioned in the plans.

## 2 Materials and methods

To provide a transparent and reproducible framework for performing this review, the authors combined the methods of a *scoping review* and an *environmental scan* [11,12].

The primary research question was: *What do state climate and health adaptation plans identify as priority needs and adaptation gaps?*

The secondary research question was: *How robust and comprehensive are the state climate and health adaptation plans in the US (according to a quality rubric developed by the authors)?*

State Climate Adaptation Plans were primarily identified through the Georgetown Climate Center’s State Adaptation Progress Tracker [13]. To assure the authors identified the most updated plans and to identify any plans missing from the Georgetown site, the following string was searched in Google as of December 2023:

(“climate change” AND “Adaptation plan” OR “health adaptation plan” OR “action plan” OR “health plan” OR “resilience plan” AND “[state of interest]”)

### 2.1 Applying inclusion and exclusion criteria

The authors screened each plan and any subsequent published updates according to the following criteria:

#### 2.1.1 Inclusion criteria

• Plans were written by a US state government or an organization such as a university, task force or nonprofit clearly identified as acting on the state government’s behalf
• Plans were entirely devoted to climate and health adaptation; or
• Plans clearly dedicated a chapter or section to climate and health adaptation (e.g., a chapter title or section that expressly mentioned health or public health); or
• If reviewing a progress or implementation update of a plan, the update identified significant new strategies and priorities

#### 2.1.2 Exclusion criteria

• Plans addressed climate change in general without specific suggestions on health adaption or solutions
• Plans addressed only health impacts of climate change or vulnerability to health impacts, without clear articulation of strategies or approaches to adaptation or resilience

The compiled plans were then independently vetted for inclusion by two authors and conflicts were resolved by a third author. Data were then extracted from the plans and entered into spreadsheets.

### 2.2 Development of a state climate and health adaptation plan quality rubric

The authors adapted the framework developed by the UN Environment Programme (UNEP) for the 2020 Adaptation Gap Reports, renaming the implementability domain as “actionable” and adding the domain of “evidence base” to assure that adaptation strategies clearly identified scientific evidence as their basis [14,15].

The authors then developed indicators for each domain specific to climate change and health adaptation as follows:

Domains

1. Comprehensiveness
2. Evidence Base
3. Inclusiveness
4. Integration
5. Actionable
6. Monitoring and evaluation

#### 2.2.1 Indicators

Comprehensiveness

1. How many exposure pathways are covered? (e.g., vector-borne diseases, wildfires, air quality, flooding/sea-level rise, extreme heat, drought)

○ 1–3 pathways=1 point, 3–5 pathways=2 points; 5+ pathways=3 points

2. Which vulnerable populations are mentioned? (e.g., older adults, children, persons with disabilities)

○ no mention=0 points, limited to age/race=2 points, extensive discussion of demographic subgroups, gender, types of disabilities=4 points

3. Which vulnerable geographies are mentioned? (e.g., coastal areas prone to flooding)

○ No mention of these issues=0 points, 1 or 2 mentions=1 point, 2+ mentions=2 points

4. How many categories of health care facility types are mentioned? (e.g., hospitals, clinics)

○ None/no mention=0 points, 1 or 2 types of facilities mentioned=1 point, 2+ types of facilitates mentioned=2 points

Evidence base

5. Does the plan refer to a previously published vulnerability or impact assessment?

○ No=0 points, Yes=2 points

6. Does the plan refer to a specific data or set of indicators of vulnerability?

○ No=0 points; only in some places=1 point, evidence-base cited throughout=2 points

7. Does the plan cite evidence or reference for adaptation actions?

○ No=0 points; only in some places=1 point, evidence-base cited throughout=2 points

Inclusiveness

8. Does the plan identify community engagement in the development of specific actions? (e.g., discussion of role of community, community organizations)

○ No=0 points; only in some places=1 point, throughout=2 points

9. Does the plan identify community engagement in monitoring and evaluation of plan?

○ No=0 points; only in some places=1 point, throughout=2 points

Integration

10. Does the plan specify actions or connections with nonprofit or other private sector organizations?

○ No=0 points, Yes=2 points

11. Are multiple state agencies representing multiple sectors involved in the plan? (e.g., horizontal integration among public health, social services, emergency management agencies, etc.)

○ No=0 points; only in some places=1 point, throughout=2 points

12. Does the plan connect to national and sub-state levels of government planning and programming? (e.g., vertical integration with counties, cities, US government, regional or multi-state planning agencies or efforts, tribal entities)

○ No=0 points; only in some places=1 point, throughout=2 points

13. Does the plan connect public health emergency preparedness, regional healthcare coalitions, and other formal health preparedness programs (horizontal health and public health sector integration)?

○ No=0 points; only in some places=1 point, throughout=2 points

Actionable

14. Is a lead agency identified (i.e., an agency is stated as being the lead or it is published under the auspices of an agency)?

○ No=0 points, Yes=2 points

15. Is a funding source identified (i.e. plan mentions who will fund actions and work discussed)?

○ No=0 points; only in some places=1 point, throughout=2 points

16. Are necessary policy changes identified (e.g., new regulations, barrier removal, incentives identified)?

○ No=0 points; only in some places=1 point, throughout=2 points

Monitoring and evaluation

17. Are metrics of success or plan outcomes identified (e.g., list of specific goals, performance measures reflecting success of plan)?

○ No=0 points; only in some places=1 point, throughout=2 points

18. Is there a timeline for evaluation?

○ No=0 points; only in some places=1 point, throughout=2 points

**Fig. 1:**
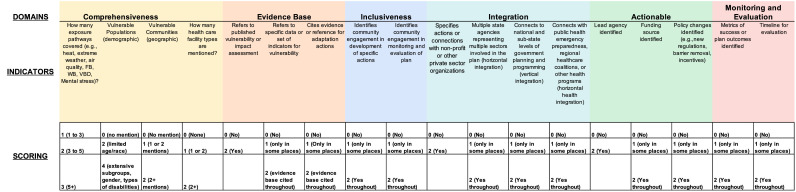
State Climate and Health Adaptation Plan Quality Rubric developed the authors, comprising six domains, eighteen unique indicators and explanation of the scoring system.

The scoring system for each indicator is as identified in Fig. 1.

### 2.3 Adaptation gap analysis

The authors coded any mentioned adaptation strategies or initiatives into the following six categories, and in addition extracted any specific adaptation gaps or challenges identified by the state plans.

1. Data/Surveillance
2. Vulnerability assessment
3. Training and capacity building
4. Funding for workforce
5. Funding for capital improvements (e.g., infrastructure or institutional creation)
6. New governance structures

## 3 Results

A total of 19 out of the 33 plans identified, with publication dates ranging from 2008 to 2020, met the authors’ inclusion criteria. Fifteen different states published climate and health adaptation plans. California published 3 updates, which were scored separately, and Maryland published one update, so that there were a total of 19 plans for the 15 states.

The number of plans and their corresponding scores in the adaptation analysis for each indicator are identified in Table 1.

**Table 1:** Listing of State Climate Change and Health Adaptation Plans along with their year of publication and total score.

State	Name of Plan	Year	Total Score
Alaska	Alaska’s Climate Change Strategy: Addressing Impacts in Alaska	2010	14
Arizona	Arizona Climate and Health Adaptation Plan 2017	2017	23
California	Safeguarding California: Public Health Sector Implementation Action Plan	2016	36
	Safeguarding California Plan: 2018 Update	2018	26
	Safeguarding California: Reducing Climate Risk −2014 Update to the 2009 CA Climate Adaptation Strategy	2014	22
	California 2009 Climate Adaptation Strategy	2009	20
Colorado	Colorado Climate Plan - State Level Policies and Strategies to Mitigate and Adapt	2015	10
Connecticut	Connecticut Climate Change Preparedness Plan	2013	19
Maryland	Comprehensive Strategy for Reducing Maryland’s Vulnerability to Climate Change Phase 2: Building societal, economic and ecological resilience	2011	15
	MD Climate Adaptation and Resilience Framework Recommendations	2020	15
Massachusetts	Massachusetts Climate Change Adaptation Report	2011	19
Michigan	Michigan Climate and Health Adaptation Program Strategic Plan Update 2016–2021	2016	22
Minnesota	Minnesota Climate and Health Strategic Plan	2016	18
New York	New York State Climate Action Plan Interim Report	2010	24
North Carolina	North Carolina 2020 Climate Risk Assessment and Resilience Plan	2020	24
Oregon	Oregon Climate and Health Resilience Plan	2017	21
Pennsylvania	Pennsylvania Climate Adaptation Planning Report: Risks and Practical Recommendations	2008	17
Washington	Preparing for a Changing Climate: Washington State’s Integrated Climate Response Strategy	2012	15
Wisconsin	Wisconsin Climate and Health Adaptation Plan	2016	27

(References to Table 1 are in Supplementary material).

### 3.1 Comprehensiveness

A total of 14 plans addressed 5 or more exposure pathways. Hazards addressed varied depending on the state and region, but they included both acute climate-related events (e.g., heat waves, wildfires and other threats to air quality, flooding, foodborne illness, vector-borne disease outbreaks) and more chronic climate-related health hazards (e.g., droughts, sea-level rise, loss of water supplies). Vulnerable populations were mentioned to varying degrees in 19 plans, with about half identifying numerous subgroups. Vulnerability in relationship to geography was far less frequently mentioned (16 out of 19 plans only had 0 to 1–2 mentions). Similarly, only 3 out of 19 plans mentioned vulnerability of health care facilities more than once or twice.

### 3.2 Evidence base

The majority of plans had no mention of a vulnerability assessment nor a specific data set that referred to indicators of vulnerability, with 13 out of 19 plans and 14 out of 19 plans receiving a score of 0 for those two indicators respectively. The majority of plans did not cite evidence or specific peer-reviewed or other references for adaptation actions. In some cases, state plans did not note previous, existing or contemporaneous analyses even if they were relevant, while in others, there simply were no relevant assessments conducted during this time period.

### 3.3 Inclusiveness

Although 11 plans did identify some degree of community engagement in the development of action items, only 3 plans incorporated engagement in monitoring and evaluation of the plans.

### 3.4 Integration

Most plans reviewed discussed connecting with community nonprofits and sectors (17/19). Nearly all achieved some degree of horizontal integration, linking the health plan with other sectors and state agencies (11/19 throughout the plan, 7/19 in some places). Similarly, nearly all plans linked the state health plan with higher and/or lower levels of government, such as national, regional, or city/county plans or policies (11/19 throughout the plan, 7/19 in some places). Additionally, the majority of plans also connected the different components of the health sector (public health, health care systems, emergency medical services and emergency management) to at least some degree (16/19).

### 3.5 Actionable

All but one state plan reviewed identified a lead agency in its development. Though some plans called for additional funding or at least mentioned funding for activities generally discussed in the plan to be implemented, the majority of plans failed to identify a specific funding source. 14 plans did not identify any funding source, while 5 plans did so only in some places or for some but not all contemplated actions. No plan included specific, identifiable funding sources for all actions throughout the plan. In addition, the majority of plans were able to identify potential policy changes to some extent, such as the need to develop new policies, guidance or regulations or efforts to bolster compliance (18/19).

### 3.6 Monitoring and evaluation

Only 2 plans had metrics of success identified throughout the plan. No plan had a comprehensive timeline for evaluation, and only 3 included limited mention of a timeline.

### 3.7 Adaptation analysis

**Fig. 2:**
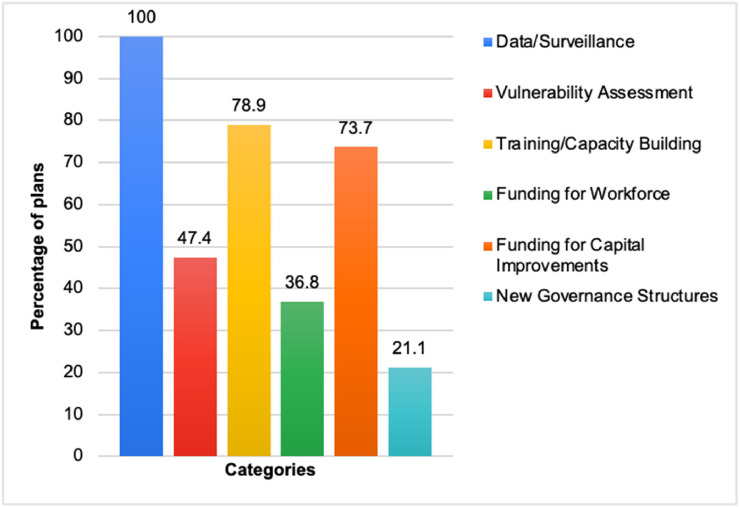
Breakdown of adaptation analysis by percentage.

All plans identified the need for data/surveillance as a priority action for states. A total of 9 plans identified a vulnerability assessment as a priority action. There were 15 plans that identified training/capacity building, 7 plans that identified funding for workforce, 14 plans that identified funding for capital improvements, and 4 plans that spoke of new governance structures in priority actions. A summary of the adaptation analysis is shown in Fig. 2

## 4 Discussion

To the best of our knowledge, this is the first published systematic assessment of quality criteria and identified priority needs and actions for State Climate and Health Adaptation Plans. The rubric created for this assessment is also unique.

In the absence of a recognized standard within the United States for state climate health adaptation plans, several analogous climate health adaptation frameworks can provide a set of baseline expectations for categories of planning to be included. The WHO framework for climate adaptation has elements in six categories for consideration at the national level [16]. The categories include *leadership and governance, financing, workforce, health information systems, essential medical products and technologies and service delivery.* HHS has published a Climate Change and Health Equity Strategy Supplement to its Agency Adaptation Plan which highlights an adaptation framework with eight components: *Data collection and integration; Research; Indicators and Measures; Workforce Training and Capacity Building; Technical Assistance and Tool Development; Innovation in Health Care Delivery; Policy Development and Funding* [17]. The major categories of these plans relevant at the state level are reflected in the six categories the authors used in the adaptation analysis to code the recommended interventions.

The lack of a standard framework for state climate and health adaptation planning and evaluation is a gap that suggests a need for more systematic collaboration among states, with national-level guidance and coordination. The fact that only 19 states in 2023 had published plans meeting inclusion criteria further underscores this need. States must also plan to ensure their work is coordinated not only with other states and the federal government but also with municipal, county, and tribal efforts.

Of the 19 plans reviewed in this analysis, there were several areas of common strengths. Most plans addressed multiple exposure pathways and identified specific populations at greater risk of health impacts. Inclusion of these issues helps ensure the plans do not overlook critical climate change-related health threats or populations that require targeted adaptation interventions. Overall, the plans consistently described integration of state health plans with other sectors and different levels of government (e.g., local, federal). Nearly all states identified multiple sectors that should participate in planning and most identified multiple state agencies as actors and stakeholders of the plan. Nearly all states demonstrated some evidence of vertical integration, and most states had at least some connection among formal health sector preparedness programs, such as regional health care coalitions, and other emergency preparedness plans.

Despite the connection to formal health sector preparedness plans, one notable weakness of the plans the authors reviewed was omission of health system impacts and resilience as critical elements. Only 3 of the 19 plans had substantive mention of healthcare systems (e.g., hospitals, clinics, health providers), and no plan showed a comprehensive or fully integrated approach to including healthcare systems in their plans. Incorporating climate adaptation planning tailored to the variety of categories of healthcare facilities, including hospitals, clinics, nursing homes, health centers, dialysis centers, community and behavioral health providers and others, is essential for protecting the most vulnerable populations from climate change health impacts.

Many plans did not identify a specific evidence base or data to support prioritization of adaptation actions. Few plans considered specific geographies within their states, such as urban heat islands or flood zones, or specific health implications for counties, tribes, towns or cities. While most states included communities to some extent in the development of their plans, there was little evidence of continued prioritization of ongoing community engagement or public input in the monitoring and evaluation of plan implementation. Lastly, the plans as a group were not highly actionable. Most plans lacked specifics for implementation, such as noting a clear funding source, describing governance, including a clear timeline, or identifying specific, clear, evaluable objectives or metrics of progress or success.

### 4.1 Adaptation gaps

The review of specified interventions revealed both common priorities and areas that were part of existing national or international health adaptation frameworks but were not prioritized by the current state plans. All plans identified a need to develop specific datasets and the evidence base to support local actions, and most identified the need for infrastructure funding, and for training and capacity building to support an effective workforce. Fewer plans discussed funding for workforce, new governance structures, or policy changes. These findings can help inform plans and programs at the national level that provide support to state-level activities. They also provide a starting point for ongoing discussions between national-level programs and state agencies to further clarify and develop priorities for adaptation actions.

### 4.2 Limitations

There are limitations of this analysis. First, the state plans reviewed varied widely in their scope. State plans varied in length and format, and timing (some plans are a decade or more old and others more recent). Plans also varied in terms of their constituents and leadership, hazards discussed, and funding and implementation details if provided. The authors’ use of a rubric and reviewing plans in tandem helped to ensure consistency in our approach.

The wide heterogeneity of scope and approach among the plans reviewed made it challenging to consistently apply the rubric in assessing plan quality. The authors attempted to make scoring criteria as objective as possible, for example counting appearances of key words, but variations in plan organization, with content sometimes appearing in multiple sections, may have limited the ability to comprehensively and accurately score each plan. The use of a consensus process among three authors for determining each plan’s score was an effort to achieve consistency and accuracy in scoring.

While every effort was made to identify published plans, it is possible the authors missed one or more plans that would have been appropriate to include in the analysis. States are continually updating and revising plans as well. The authors also note that some states may be engaged in important climate and health-related work but are doing so without a formal plan or at least a plan that has not yet been published. Therefore, this analysis may not reflect missed or updated plans.

The lack of a consensus template or quality criteria for state level climate and health adaptation plans also presented challenges to this study. The United States healthcare and public health systems have unique complexities in governance, funding, and scale that limit the applicability of adaptation planning guidance that exists for other parts of the world. While the authors based the scoring rubric used in this analysis on existing published adaptation frameworks and guidance, it was not developed through a broader consensus-based process. There may be disagreement about categories or approaches to determining quality. The authors hope that this initial effort to codify components and quality of state-level climate and health adaptation plans leads to such a broader consensus-based process and wider adoption of a common framework and approach.

## 5 Conclusion

The analysis in this review is the first attempt the authors are aware of at a comprehensive assessment of state level climate and health adaptation plans at the state level. As of 2023, only 19 of 50 states had developed climate health adaptation plans that met inclusion criteria, embedded in either their state adaptation plans or as stand-alone state health adaptation plans. Furthermore, there is substantial variation in comprehensiveness and quality among the 19 plans. States took varying approaches to addressing issues such as funding, research and evidence base, and equity. Many states also have yet to include actionable metrics and a timeline for implementation or a clear approach to updating plans that in some cases already are several years old. Notably, most states did not prioritize health system and health sector vulnerabilities in their plans. As the recent tragic experiences with hurricanes Helene and Milton have demonstrated, health sector vulnerabilities can have devastating local, regional and national consequences in a setting of extreme weather disasters [18,19].

The authors believe that this analysis can serve two purposes: it provides a snapshot of the current state of the art of state-level adaptation planning as well as a framework for developing consensus guidance and templates; and it helps inform national-level adaptation planning by identifying shared themes and priorities for state-level adaptation efforts. By identifying strengths and gaps in the current set of state-level adaptation plans, this analysis can help guide ongoing state efforts to enhance resilience to climate-related health hazards and initiate adaptation measures. In addition, by identifying shared priorities and needs at the state level, this analysis can also help inform future national-level priorities and actions to support states in their climate adaptation planning

## CRediT authorship contribution statement

**Katharine Weber:** Writing – review & editing, Writing – original draft, Visualization, Methodology, Investigation, Formal analysis, Data curation, Conceptualization. **Aparna Bole:** Writing – review & editing, Project administration. **John Balbus:** Writing – review & editing, Writing – original draft, Validation, Supervision, Project administration, Methodology, Investigation, Formal analysis, Data curation, Conceptualization.

## Declaration of competing interest

The authors declare that they have no known competing financial interests or personal relationships that could have appeared to influence the work reported in this paper.

## Appendix Supplementary materials

Image, application 1

## Footnotes

Supplementary material associated with this article can be found, in the online version, at doi:10.1016/j.joclim.2025.100593.
