# Supplementary material for: Climate change and health: An assessment of state level adaptation plans
Source: J Clim Chang Health. 2025 Oct 22;26:100593. doi: 10.1016/j.joclim.2025.100593 (PMC12851340; doi:10.1016/j.joclim.2025.100593)
Supplement: Supplementary file 1 [file mmc1.pdf]

References:

1. Adaptation Advisory Group. Alaska's Climate Change Strategy: Addressing Impacts in Alaska. Alaska Department of Environment Conservation; 2010. Available from: https://www.cakex.org/sites/default/files/documents/aag_all_rpt_27jan10.pdf

2. Roach M, Barrett E, Brown HE, Dufour B, Hondula DM, Putnam H, et al. Climate and Health Adaptation Plan | 2017. Phoenix (AZ): Arizona DHS; 2017. Available from: https://www.azdhs.gov/documents/preparedness/epidemiology-disease-control/extreme-weather/pubs/arizona-climate-health-adaptation-plan.pdf
3. CDPH. Safeguarding California: Implementation Action Plans Public Health Sector Plan. Sacramento (CA): CDPH; 2016. Available from: https://www.adaptationclearinghouse.org/resources/safeguarding-california-public-health-sector-implementation-action-plan.html
4. California Natural Resources Agency. Safeguarding California: Implementation Action Plans. California Natural Resources Agency; 2016. Available from: https://www.adaptationclearinghouse.org/resources/safeguarding-california-implementation-action-plans.html
5. California Natural Resources Agency. Safeguarding California: Reducing Climate Risk – 2014 Update to the 2009 CA Climate Adaptation Strategy. California Natural Resources Agency; 2014. Available from: https://www.adaptationclearinghouse.org/resources/safeguarding-california-reducing-climate-risk-2014-update-to-the-2009-ca-climate-adaptation-strategy.html
6. California Natural Resources Agency; State of California. 2009 California Climate Adaptation Strategy. California Natural Resources Agency; 2014. Available from: https://www.adaptationclearinghouse.org/resources/california-2009-climate-adaptation-strategy.html
7. Colorado Energy Office; Colorado Resiliency and Recovery Office; Department of Agriculture; Department of Local Affairs; Department of Natural Resources; Department of Public Health and the Environment; et al. Colorado Climate Plan – State Level Policies and Strategies to Mitigate and Adapt (2015). CDOT; 2015. Available from: https://www.adaptationclearinghouse.org/resources/colorado-climate-plan-state-level-policies-and-strategies-to-mitigate-and-adapt-2015.html
8. Governor's Steering Committee on Climate Change Adaptation Subcommittee. Connecticut Climate Preparedness Plan. CT DEEP; 2011 [cited 2025 Sep 2]. Available from: https://portal.ct.gov/-/media/deep/climatechange/connecticutclimatepreparednessplan2011pdf.pdf
9. Maryland Commission on Climate Change; University of Maryland Center for Environmental Science. Comprehensive Strategy for Reducing Maryland's Vulnerability to Climate Change Phase II: Building Societal, Economic, and Ecological Resilience [Internet]. UMCES; 2011. Available from: https://ian.umces.edu/site/assets/files/10938/comprehensive-strategy-for-

reducing-marylands-vulnerability-to-climate-change-phase-ii-building-societal-economic-and-ecolo.pdf
10. Maryland Department of the Environment; Maryland Commission on Climate Change. Maryland Climate Adaptation and Resilience Framework: Recommendations 2021-2030. MDE; 2020. Available from: https://mde.maryland.gov/programs/air/ClimateChange/MCCC/Documents/MD%20Climate%20Adaptation%20and%20Resilience%20Framework%20Recommendations.pdf
11. Commonwealth of Massachusetts. Massachusetts Climate Change Adaptation Report. Commonwealth of Massachusetts; 2011. Available from: https://www.adaptationclearinghouse.org/resources/massachusetts-climate-change-adaptation-report.html
12. Cameron LL, Ferguson A, Karner C. Michigan Climate and Health Adaptation Program (MICHAP) Strategic Plan Update: 2016-2021. Michigan Department of Health and Human Services; 2016. Available from: https://www.michigan.gov/-/media/Project/Websites/mdhhs/Folder2/Folder16/Folder1/Folder116/MDCH_climate_change_strategicPlan_final_1-24-2011_.pdf?rev=d80f9db32c9649538a4f43668a9cecd7
13. Minnesota Department of Health. The Minnesota Climate & Health Strategic Plan August 2016-2021. St. Paul (MN): MDH; 2020 [cited 2025 Sep 2]. Available from: https://www.health.state.mn.us/communities/environment/climate/docs/strategicplan.pdf
14. New York State Climate Action Council. Chapter 1: Introduction to Climate Change Adaptation. In: Climate Action Plan Interim Report. New York State Climate Action Council; 2010. Available from: https://extapps.dec.ny.gov/docs/administration_pdf/irchap1.pdf
15. North Carolina Department of Environmental Quality. North Carolina Climate Assessment and Resilience Plan. NCDEQ; 2020. Available from: https://www.adaptationclearinghouse.org/resources/north-carolina-2020-climate-risk-assessment-and-resilience-plan.html
16. OCCRI. The Oregon Climate Change Adaptation Framework. Oregon Climate Change Research Institute; 2010. Available from: https://www.adaptationclearinghouse.org/resources/the-oregon-climate-change-adaptation-framework.html
17. PDEP. Pennsylvania Climate Adaptation Planning Report: Risks and Practical Recommendations. Pennsylvania Department of Environmental Protection; 2011. Available from: https://www.adaptationclearinghouse.org/resources/pennsylvania-climate-adaptation-planning-report-risks-and-practical-recommendations.html
18. Adelsman H, Ekrem J. Preparing for a Changing Climate: Washington State’s Integrated Climate Response Strategy. Washington State Department of Ecology; 2012. Available from: https://www.adaptationclearinghouse.org/resources/preparing-for-a-changing-climate-washington-state-s-integrated-climate-response-strategy.html

19. Wisconsin DHS. Wisconsin Climate and Health Adaptation Plan. Wisconsin Department of Health Services; 2016. Available from: https://www.dhs.wisconsin.gov/publications/p01447.pdf
